# Influence of Simulated Oral Conditions on Different Pretreatment Methods for the Repair of Glass-Ceramic Restorations

**DOI:** 10.3290/j.jad.b2701717

**Published:** 2022-03-01

**Authors:** Benedikt Höller, Renan Belli, Anselm Petschelt, Ulrich Lohbauer, José Ignacio Zorzin

**Affiliations:** a Doctoral Student, Dental Clinic 1, Restorative Dentistry and Periodontology, University Clinic Erlangen, Friedrich Alexander University, Erlangen-Nürnberg, Germany. Idea, hypothesis, experimental design, performed the experiments and statistical evaluation, and wrote the manuscript in fulfilment of the requirements for the degree “Dr. med. dent.”; b Research Associate, Dental Clinic 1, Restorative Dentistry and Periodontology, University Clinic Erlangen, Friedrich Alexander University Erlangen-Nürnberg, Germany. Proofread the manuscript, contributed to the experimental design, contributed substantially to discussion.; c Professor, Dental Clinic 1, Restorative Dentistry and Periodontology – University Clinic Erlangen, Friedrich Alexander University Erlangen-Nürnberg, Germany. Proofread the manuscript, contributed substantially to discussion.; d Associate Professor, Dental Clinic 1, Restorative Dentistry and Periodontology, University Clinic Erlangen, Friedrich Alexander University Erlangen-Nürnberg, Germany. Idea, hypothesis, experimental design, proofread the manuscript, contributed substantially to discussion.; e Research Associate, Dental Clinic 1, Restorative Dentistry and Periodontology, University Clinic Erlangen, Friedrich Alexander University Erlangen-Nürnberg, Germany. Idea, hypothesis, experimental design, performed the statistical evaluation, contributed substantially to the manuscript.

**Keywords:** self-etching glass-ceramic primer, grit blasting, silanization, lithium-disilicate ceramic, tensile bond strength, thermocycling

## Abstract

**Purpose:**

The present study investigated the influence of simulated intraoral conditions (increased temperature and humidity) on two different surface pretreatment methods to repair a lithium-disilicate glass-ceramic (LDS).

**Materials and Methods:**

A total of 540 rectangular lithium-disilicate glass-ceramic bars were manufactured (3 x 7 x 9 mm; IPS e.max CAD, Ivoclar Vivadent). Further specimen preparation was performed in an incubator with controlled relative humidity (RH) and temperature to simulate three different environmental settings: laboratory conditions (LC, n = 180, 23°C, 50% RH), rubber-dam conditions (RC, n = 180, 30°C, 50% RH) or oral conditions (OC, n = 180, 32°C, 95 ± 5% RH). One-third of the bars under each condition (n = 60) were grit blasted (GBL) with alumina (35 µm at 1 bar pressure for 10 s and a working distance of 4 ± 1 cm) and primed (60 s, Monobond Plus, Ivoclar Vivadent). Another third (n = 60) were pretreated with a self-etching glass-ceramic primer (MEP, Monobond Etch & Prime, Ivoclar Vivadent). One group without surface pretreatment (n = 60, NoPT) served as a control. All pretreated surfaces were coated with Heliobond (Ivoclar Vivadent). Two bars from the same pretreatment method were luted perpendicular to each other with a resin composite to form a square adhesion area of 9 mm^2^ (TetricEvo Ceram, Ivoclar Vivadent), and light cured for 20 s on each side (1200 mW/cm^2^, Bluephase 20i, Ivoclar Vivadent). All specimens were stored for 24 h in distilled water at 37°C. Half of the specimens from each environmental setting and pretreatment method (n = 15) were thermocycled (TC, 5000 cycles, 5/55°C, 30-s dwell time), and tensile bond strength (TBS) testing was performed for all groups using an x-bar rope-assisted set-up. Data were statistically analyzed using two-way ANOVA (α = 0.05) with Bonferroni adjustment.

**Results:**

Regardless of the environmental and storage conditions (24 h or TC), MEP showed a significantly higher mean TBS than GBL. A decrease in TBS was recorded in specimens under OC compared to RC and LC for both pretreatment methods independent of the storage condition. No significant difference in mean TBS was found between RC and LC within the MEP pretreatment group for the 24 h stored and thermocycled specimens. For all MEPs and GBLs, TC reduced the mean TBS in all environmental conditions. The NoPT groups showed no adhesion regardless of environmental or storage conditions.

**Conclusions:**

Increased temperature and high humidity significantly reduced TBS. However, MEP was less sensitive to environmental influences than GBL, which makes it a promising candidate for intraoral ceramic repair. These findings suggest that clinical intraoral repair of lithium-disilicate glass-ceramics should be performed using a rubber-dam, primarily when using GBL.

Increasing demand for esthetic and biocompatible materials has led to a higher interest in ceramic restorations in restorative and prosthetic dentistry over the past decades.^36^ Accordingly, several ceramic systems based on different compositions were introduced into dental clinical practice.^31^ Glass-ceramic composites reinforced with lithium disilicate enjoy great popularity, due to their high esthetic quality and mechanical characteristics.^26,51^ Because of the brittleness of ceramic materials, fracture and chipping of ceramics is a common clinical complication.^55,73^ Chipping may occur from material fatigue, manufacturing flaws, lack of adhesive bond, inappropriate restoration design or occlusal stress situations, such as parafunctional occlusal contacts and interferences.^24,59^ Fractured areas represent an esthetic and functional problem, which makes restoration replacement generally indispensable. These replacements require several clinical sessions and extensive additional costs.^50^

The intraoral repair of ceramic fractures is a minimally invasive clinical treatment option in most cases, and it is commonly performed using resin composites.^24,50^ To achieve long-term adhesion and success with resin composite repair, pretreatment of the ceramic surface is essential.^23^ The fractured surface is thoroughly cleaned, pretreated to increase micromechanical retention and silanized to chemically activate the hydrophilic silicate ceramic for coupling to a hydrophobic luting composite.^33,43-45^ Several repair systems based on different pretreatment techniques have been reported, but none of them create such strong and long-lasting adhesion as does the extraoral gold-standard procedure using hydrofluoric acid (HF) etching with subsequent silanization.^1,11,16,44,54,57^ Although HF (2%-10%) is potentially toxic, may cause severe acid burns with serious damage to body tissues, and should be avoided for intraoral use, it is a frequently applied pretreatment method for ceramic repair in dental clinical practice.^33,38,46,54^ An alternative technique for intraoral repair, which does not involve the risk of acid burns, is pretreatment of the surface by grit blasting with alumina powder, also known as air abrasion, followed by silanization.^28,50^ Using this technique, surface cleaning and roughening for micromechanical retention is obtained by propelling small Al_2_O_3_ particles (up to 110 µm) with pressurized air onto the ceramic surface.^44^ Although this method has less toxic potential than HF, eye and breathing protection are essential to avoid dust inhalation, and a rubber-dam is needed to prevent soft tissue damage and surgical emphysema.^14,18^ Considerable weakening of bond strength may occur due to contamination with Al_2_O_3_ particles or surface damage from the grit-blasting technique itself.^10,44^ As a simplified one-step pretreatment technique, a self-etching glass-ceramic primer (Monobond Etch & Prime, Ivoclar Vivadent; Schaan, Liechtenstein) that contains ammonium polyfluoride as an etching agent and trimethoxypropyl methacrylate for silanization was introduced on the market.^9,19,65^ This primer is brushed onto the glass-ceramic surface, rinsed with water after a 40-s reaction time, and air dried for 10 s.^71^ During air drying, the silane chemically activates the hydrophilic ceramic surface for adherence to a hydrophobic luting composite.^43^ The chemical compounds of MEP are considered less harmful than HF, but they are potentially toxic.^3,39^

Several laboratory studies and a few clinical case reports have evaluated the bond strength of the new self-etching primer. Some laboratory studies demonstrated lower bond strengths for several application modes of MEP compared to the gold-standard HF on different bonding materials to lithium-disilicate glass-ceramics.^8,19,26,54^ In contrast, other studies showed a higher or comparable Weibull modulus or bond strength of MEP-pretreated glass-ceramics compared to HF followed by silane.^13,20-21,25,56,60,63^ Based on these findings, some authors recommend MEP as an alternative to HF and silane.^2,41^ Compared to other universal adhesives, MEP showed high bond strength performance.^35,69^ Notably, two recently published case reports revealed promising esthetic and mechanical long-term performances of MEP.^62,64^ Lyann et al^42^ additionally demonstrated that MEP effectively removed saliva contamination and enhanced the resin bond strength to lithium-disilicate glass-ceramics. The simplified one-step application of MEP and the presumed lower hazard to body tissues support this material as a clinically promising alternative pretreatment method for intraoral ceramic repair to overcome the limitations of conventional pretreatment methods using HF.^2,68,72^

For in vitro tensile bond strength tests, specimens are principally prepared under defined environmental conditions in the laboratory, which are generally 23 ± 2°C and 50 ± 5% relative humidity (RH).^4^ However, oral environmental conditions are characterized by temperatures above 30°C and approach a saturated humidity up to 95% RH.^30^ When using dry-field techniques, the temperature and above all RH may be significantly reduced to approximately 50%.^27^ Temperature influences the viscosity and reactivity of resin-based materials, and increasing RH significantly reduces the adhesive properties of different bonding systems.^12,27,30,47,53^ To the best of our knowledge, pretreatment methods for the intraoral repair of glass-ceramics, such as grit blasting or use a self-etching glass-ceramic primer, have only been assessed under laboratory environmental conditions.^22,43^

The present study compared the resin composite tensile bond strengths (TBS) to lithium-disilicate glass-ceramic using two different pretreatment methods, grit blasting (GBL) and a self-etching glass-ceramic primer (MEP), under different environmental conditions (laboratory, rubber-dam, or oral conditions) after 24 h and thermocycling (TC). The first null hypothesis was that the combination of each pretreatment method (MEP or GBL) and TC or 24 h storage would not influence TBS under the same environmental conditions (LC, RC, or OC). The second working hypothesis was that the combination of environmental conditions (LC, RC, or OC) and storage conditions (24 h or TC) would not influence TBS within the same pretreatment method (GBL or MEP).

## Materials and Methods

The materials used, along with their batch numbers and composition, are listed in Table 1.

**Table 1:** Materials used, manufacturers, batch numbers, and composition as per manufacturer information

Material	Manufacturer and Batch No.	Composition
Monobond Etch and Prime, self-etching glass-ceramic primer	Ivoclar Vivadent; Schaan, Liechtenstein V50918	Tetrabutylammonium dihydrogen trifluoride, trimethoxylpropyl methacrylate, methacrylated phosphoric acid ester, butanol, water, colorant
Monobond plus, universal primer for all types of restorative materials	Ivoclar Vivadent W95471	Ethanol, silane methacrylate, phosphoric acid methacrylate, sulphide methacrylate
Heliobond, non-solvated bonding resin	Ivoclar Vivadent W28595	Bisphenol A-glycidyl methacrylate (bis-GMA) 50–100%vol Triethylene glycol dimethacrylate (TEG-DMA) 25–50%vol
Tetric Evo Ceram A3, nano-hybrid condensable resin composite	Ivoclar Vivadent W93700	Dimethacrylates Fillers containing barium glass, ytterbium trifluoride, mixed oxide and copolymers Additives, initiators, stabilisers and pigments
IPS e.max CAD LT A2/C14, lithium-disilicate glass-ceramic	Ivoclar Vivadent W93237OV	SiO_2_, Li_2_O, K_2_O, P_2_O_5_, ZrO_2_, ZnO, Al_2_O_3_, MgO, coloring oxides

### Environmental Conditions for Specimen Preparation

All specimen preparations, including pretreatment procedures and luting, were performed under different previously published environmental conditions with specific temperatures and relative humidity values (RH).^27^ For specimen preparation under laboratory conditions (LC), the air conditioning system was set to 23 ± 1°C, and the RH was 50 ± 5%. Specimen preparation under simulated rubber-dam (RC, 30 ± 1°C, RH 50 ± 5%) and oral conditions (OC, 32 ± 1°C, RH 95 ± 5%) was performed in a specially modified incubator hood (Bühler TH 15 Incubator, Edmund Bühler; Hechingen, Germany) that permitted specimen preparation inside. The humidity inside of the incubator was adjusted to the desired level with a water bowl connected to a bath circulator (40°C water temperature, Haake D8, Haake Messtechnik; Karlsruhe, Germany) equipped with an ultrasonic water fogger (Fogger, 100 LED white, Selinger; Villingen, Germany). For all environmental conditions (LC, RC, OC), temperature and humidity were continuously monitored using a hygrometer (TFH 620, RH range: 0%-100%, temperature range: 0°C-60°C, Ebro Electronic; Ingolstadt, Germany).

### Specimen Preparation

For specimen preparation, lithium-disilicate glass-ceramic CAD/CAM blocks (LDS, IPS e.max CAD for Cerec and InLab LT A2/C14, Ivoclar Vivadent) were cut into 540 rectangular bars of 3 mm width, 7 mm height and 9 mm length under profuse water cooling (IsoMet Low Speed Saw, Buehler; Lake Bluff, IL, USA) and cup-ground (MPS 2120, G&N; Erlangen, Germany). All bars were crystallized as recommended by the manufacturer (heating rate t_1_ = 30°C/min, firing temperature T_1_=850°C, holding time H_1_ = 10 min; Vacumat 4000, Vita Zahnfabrik; Bad Säckingen, Germany).

For the glass-ceramic grit blasting pretreatment groups (GBL), 180 LDS bars were grit blasted with alumina (35 µm, No. 280, Hafra; Aßling, Germany) at 1 bar pressure for 10 s and a working distance of 4 ± 1 cm. The grit-blasted surfaces were cleaned with water spray and dried with oil-free air. The cleaned bars (n = 60 per environmental condition) were left to temper under the respective environmental conditions (15 min at LC, RC or OC) to prevent extensive water condensation on the bonding surfaces. After tempering, the grit-blasted surfaces were silanized (60 s, Monobond Plus, Ivoclar Vivadent) under each condition (LC, RC, OC) before the luting procedure.

For the self-etching glass-ceramic primer pretreatment groups (MEPs), a total of 180 LDS bars (n = 60 per environmental condition) were left to temper under the respective environmental conditions (15 min at LC, RC or OC) to prevent extensive water condensation on the bonding surfaces. The bars were pretreated with the self-etching glass-ceramic primer (Monobond Etch & Prime, Ivoclar Vivadent) as recommended by the manufacturer. This consisted in rubbing the self-etching glass-ceramic primer onto the surface of the specimens using a small brush (Roundtip Applicator, Henry Schein; Melville, NY, USA) for 20 s, letting it react for 40 s, followed by rinsing with water and drying with oil-free air for 10 s before the luting procedure.

For the control groups (NoPT), a total of 180 LDS bars (n = 60 per environmental condition) were left to temper under the respective environmental conditions (15 min at LC, RC or OC) to prevent extensive water condensation on the bonding surfaces. These bars received no pretreatment.

Under the corresponding environmental conditions (LC, RC, or OC), the luting procedure of all specimens (GBL, MEP, and NoPT) was performed to produce the final TBS test specimens (n = 15 per group). The pretreated surface of each bar was coated with an unfilled resin (Heliobond, Ivoclar Vivadent) and thinned with oil-free airflow. Two bars from the same pretreatment group were luted together perpendicular to each other with a nanohybrid condensable resin composite (Tetric EvoCeram A3, Ivoclar Vivadent) under a constant pressure of 10 N to form test specimens with a square bonded area of approximately 9 mm^2^. Resin composite excess was carefully removed using a small brush (Roundtip Applicator, Henry Schein). The resin composite was light cured from each side of the luting gap (20 s, 1200 mW/cm^2^, high power mode, Bluephase 20i equipped with Light Probe 10>8 mm Black, Ivoclar Vivadent). All specimens were stored for 24 h in distilled water at 37°C. After storage, half of the specimens from each group (n = 15) were thermocycled (TC, 5000 cycles, 5/55°C, 30-s dwell time, SD Mechatronik; Feldkirchen-Westerham, Germany).

### Tensile Bond Strength Testing

**Fig 1:**
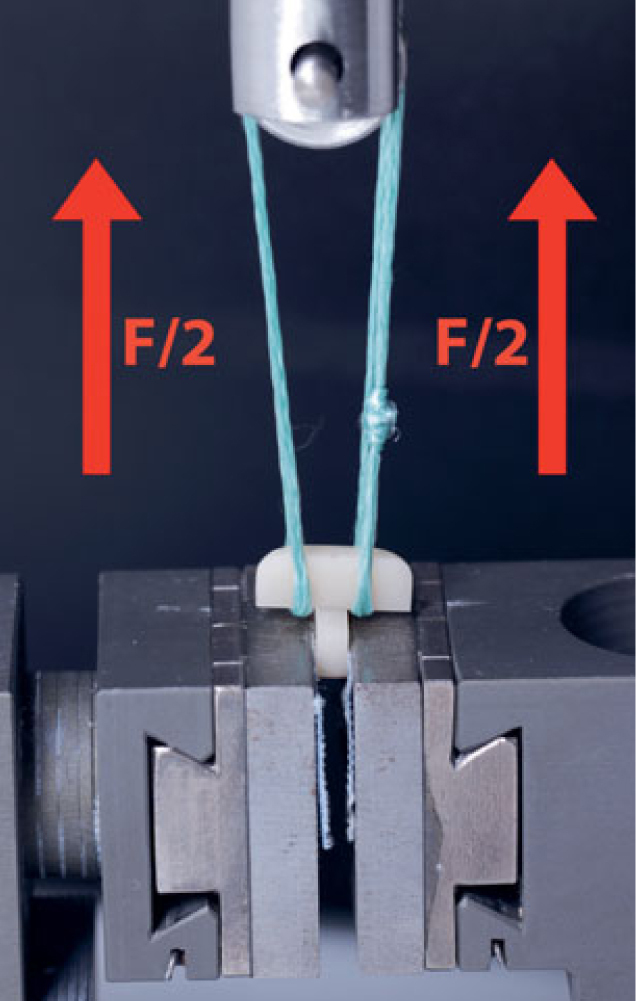
X-bar rope setup for TBS testing. The perpendicularly luted test specimen is mounted on the load frame, with the lower part held in a screw grip. A low-compliance rope is used to pull the other part of the specimen up during the TBS testing using a pulley. Applied tensile force is visualized as red arrows.

Tensile bond strength (TBS) testing was performed using an x-bar rope-assisted set-up developed by Lohbauer et al.^37^ Measurements were performed in a universal testing machine (crosshead speed of 1 mm/min, Z 2.5, Zwick-Roell; Ulm, Germany) with a low-compliance rope on a pulley (Dyneema SK75, DSM Dyneema; Stanley, NC, USA) to hold the sample and ensure a balanced force application of F/2 for each side of the upper specimen (Fig 1). The TBS was calculated by dividing the maximum applied load at fracture (N) by the area of the adhesive interface of each specimen (mm^2^) after testing, and measured under a stereomicroscope (Stemi SV6, Carl Zeiss Mikroskopie; Jena, Germany) with digital imaging analysis software (Axiovision 4.8, Carl Zeiss Mikroskopie). Specimens that failed before measurements were included as pre-test failures (PTFs) with an TBS of 0 MPa.

### Statistical Analysis

All data calculations and statistical analyses were performed using IBM SPSS 26.0 (IBM; Armonk, NY, USA). Residual analysis was performed to test for the assumptions of the two-way ANOVA. Normal distributions were assessed using Shapiro-Wilk’s test of normality (p>0.05), and homogeneity of variances was evaluated using Levene’s test for each cell of the design. A two-way ANOVA (α = 0.05) was performed to examine whether the combination of pretreatment method (MEP or GBL) and storage condition (24 h or TC) affected TBS within the same environmental conditions (LC, RC, OC). The effect of the combination of environmental conditions (LC, RC, OC) and storage conditions (24 h or TC) on TBS within the same pretreatment method was analyzed. Statistical analysis of simple main effects for each combination was determined using a Bonferroni adjustment and pairwise comparisons for each simple main effect (α = 0.025).

## Results

**Fig 2:**
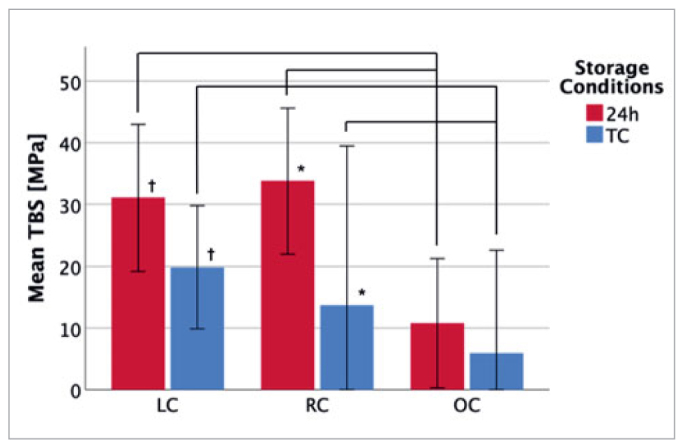
MEP group. Mean TBS of MEP-pretreated specimens under different environmental and storage conditions. * and † indicate significant differences (p < 0.025) between storage condition groups; connecting lines indicate significant differences (p < 0.025) between environmental condition groups. Statistically significant differences were observed between samples stored for 24 h and thermocycled in the LC and RC groups. The mean TBS of samples under LC and RC was significantly higher compared to the OC group, independent of the storage conditions. TBS: tensile bond strength; LC: laboratory conditions; RC: rubber-dam conditions; OC: oral conditions; MEP: Monobond Etch & Prime; GBL: grit blasting; 24 h: stored for 24 h in distilled water; TC: thermocycling.

**Fig 3:**
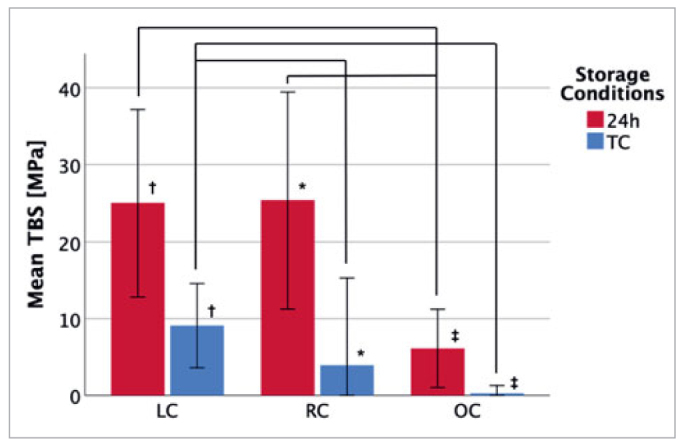
GBL group. Mean TBS of GBL-pretreated specimens under different environmental and storage conditions. *, †, and ‡ indicate significant differences (p < 0.025) between storage condition groups; connecting lines indicate significant differences (p < 0.025) between environmental condition groups. A statistically significant difference between 24 h and TC for all environmental conditions was found. For the specimens stored for 24 h, the mean TBS was significantly higher in the LC and RC groups compared to samples under OC. Thermocycled samples exhibited significantly different mean TBS between LC and RC and between LC and OC. TBS: tensile bond strength; LC: laboratory conditions; RC: rubber-dam conditions; OC: oral conditions; MEP: Monobond Etch & Prime; GBL: grit blasting; 24 h: stored for 24 h in distilled water; TC: thermocycling.

**Fig 4:**
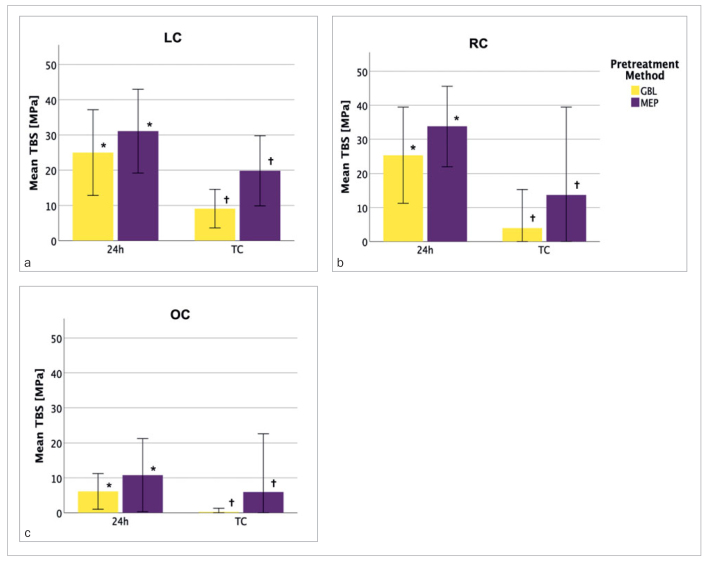
Mean TBS of GBL and MEP pretreated samples under different storage conditions for LC (a), RC (b) and OC (c). * and † indicate significant differences (p < 0.025) between GBL (yellow) and MEP (purple) pre-treated groups. Only statistically significant differences between pretreatment methods are considered in this figure. MEP pretreated specimens showed a significantly higher mean TBS for both storage conditions (TC, 24 h) and under LC (a), RC (b) and OC (c). Simulation of oral conditions strongly reduced the mean TBS (c). TBS: tensile bond strength, LC: laboratory conditions, RC: rubber-dam conditions, OC: oral conditions, MEP: Monobond Etch & Prime; GBL: grit blasting; 24 h: stored for 24 h in distilled water; TC: thermocycling.

The mean resin composite TBS to lithium-disilicate glass-ceramic for all groups tested is summarised in Table 2 and Figs 2 to 4.

**Table 2:** Mean tensile bond strengths and standard deviations [], followed by the number of pre-test failures, of the tested pretreatment methods under different environmental conditions after 24-h storage and thermocycling

	TBS (MPa)					
	LC		RC		OC	
	24 h	TC	24 h	TC	24 h	TC
MEP	31.1 [5.9] 0	19.8 [5.0] 0	33.8 [5.9] 0	13.7 [5.9] 0	10.8 [5.2] 0	5.9 [8.4] 1
GBL	25.0 [6.1] 0	9.1 [2.7] 0	25.3 [7.0] 0	3.9 [5.7] 3	6.1 [2.6] 0	0.3 [0.5] 11
NoPT	2.9 [2.6] 5	0 [0.0] 15	0.2 [0.4] 10	0 [0.1] 14	0.1 [0.1] 6	0 [0.0] 15

The number of pre-test failures was included in the statistic analysis with a tensile bond strength of 0 MPa. TBS: tensile bond strength; LC: laboratory conditions; RC: rubber-dam conditions; OC: oral conditions; MEP: Monobond Etch & Prime; GBL: grit blasting; 24 h: stored for 24 h in distilled water; TC: thermocycling.

To perform two-way ANOVA, the assumptions of normal distribution and homogeneity of variances were tested. If one of these assumptions was not fulfilled, two-way ANOVA was performed because of its robustness when group sizes are equal (Table 2).

The measured TBS in all NoPT groups was zero or nearly zero (Table 2). Therefore, the data of the NoPT groups were excluded from further statistical analyses.

### Influence of Pretreatment Method and Storage Conditions within Different Environmental Conditions

Pairwise comparisons revealed significantly higher mean TBS in MEP-pretreated specimens than GBL-pretreated samples under every environmental condition and storage condition (p < 0.025, Fig 4).

### Influence of Environmental and Storage Conditions within the Two Pretreatment Methods

#### Influence within the MEP group

The effects of environmental and storage conditions on MEP-pretreated specimens were analyzed. Samples under OC exhibited significantly lower mean TBS than specimens treated under LC or RC for thermocycled and 24-h stored specimens (Fig 2). Notably, no statistically significant difference in the mean TBS was observed between the LC and RC groups for each storage condition (24 h or TC). Comparison of thermocycled and 24-h stored specimens within the MEP pretreatment group revealed significantly different mean TBS for samples treated under LC (p < 0.001) and RC (p < 0.001), with samples stored for 24 h exhibiting 11.25 MPa and 20.12 MPa higher mean TBS (mean TBS24h – mean TBSTC) than thermocycled samples, respectively. No significant difference was found between the OC samples (Fig 2).

#### Influence within the GBL group

Analogous to the MEP pretreatment group results, the environmental condition OC resulted in a significant decrease in mean TBS compared to LC and RC for both storage conditions. The effect of OC on the mean TBS was more pronounced in the 24-h storage group and showed differences of 18.90 MPa and 19.24 MPa for LC and RC, respectively (Fig 3). A nearly complete loss of bond stability occurred in GBL-pretreated and thermocycled specimens under OC, with a mean TBS of 0.30 MPa, where 11 of the blocks broke apart before the TBS testing could be performed (Table 2). Notably, no significant difference was observed in mean TBS between LC and RC for the 24-h group, but significantly different mean TBS scores were recorded for LC and RC after thermocycling (Fig 3). The mean TBS for samples stored for 24 h was significantly higher than thermocycled specimens under all environmental conditions.

## Discussion

**Fig 5:**
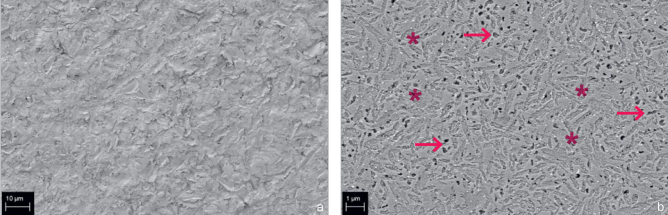
Representative SEM images of the surface texture of the glass-ceramic (IPS e.max CAD) after different pretreatment methods. a) GBL. b) MEP (Monobond Etch & Prime) without silane layer. The resulting etching pattern is less pronounced than GBL and occurs at the phase boundaries (*) and the pre-existing pores (arrows).

Pairwise comparison revealed that MEP-pretreated specimens exhibited significantly increased TBS compared to GBL-pretreated samples for three different environmental conditions (LC, RC or OC) and two defined storage conditions (24 h or TC). The first working hypothesis, that the combination of pretreatment method and storage condition would have no significant influence on TBS within the same environmental condition, was rejected. Although grit blasting is an effective method for cleaning and creating a rough microretentive surface (Fig 5a), it also induces subsurface damage to the ceramic, which makes it more susceptible to stress and prone to crack propagation.^23,66,74^ Therefore, a lower TBS compared to MEP was induced. In contrast, pretreatment with the glass-ceramic primer in the MEP groups resulted in a smooth, etched surface (Fig 5b), and phase boundaries and voids were areas of dissolution.^48-49,74^ Similar etching patterns were reported for ammonium bifluoride etchants.^17^ Mild etching presumably deteriorates the mechanical resistance of the ceramic part of the adhesive interface to a lesser extent than does GBL. Improved chemical reactivity for silane bonding by promoting hydroxyl formation on the ceramic surface, which occurs with hydrofluoric acid etching, may also be proposed for the MEP groups, where etching is produced by ammonium polyfluoride and leads to stable silanization and reliable adhesion.^45^ Conversely, alumina particles can remain on the roughened ceramic surface after grit blasting, which creates alumina-silane bonds after silanization with lower hydrolytic stability than ceramic-silane bonds.^32^ The mechanism mentioned above may explain the inferior TBS of the GBL compared to the MEP groups. GBL pretreatment is a two-step procedure comprised of grit blasting and subsequent silanization. Underhill et al^70^ examined the curing of silane at temperatures between 20°C and 40°C and relative humidity from 40% to 90% and demonstrated that increasing environmental humidity negatively affected silanization.^70^ Those authors attributed this result to an enhanced remnant amount of water disrupting silane bonds to the adhesion substrate. These findings are consistent with our results, where the increased environmental humidity may also have impaired the silanization process of GBL specimens. However, the influence of increased temperature and humidity values on the pretreatment methods MEP or GBL in our study should not be translated to materials other than the materials under investigation. Notably, a no pretreatment group (NoPT), where the specimen surface was left untreated, with no chemical or mechanical interlocking between the resin bond and ceramic material, served as the control group. The significant influence of different environmental conditions could not be discriminated because most of these specimens broke apart before measurement.

**Fig 6:**
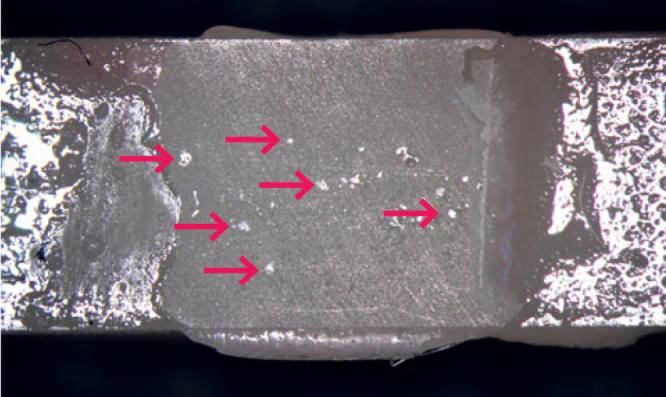
Representative stereomicroscopic image of the luting interface of a GBL specimen manufactured under OC after 24-h storage and TBS testing. Groups of voids in the adhesive interface (arrows) likely resulted from water droplets due to higher humidity levels under OC.

The present work also investigated whether the combination of environmental (LC, RC, or OC) and storage conditions (24 h or TC) influenced the mean TBS in the MEP or GBL pretreatment group. Specimens in the GBL and MEP groups manufactured under OC exhibited a significant decrease in TBS compared to specimens manufactured under LC and RC, independent of storage conditions. Therefore, the second working hypothesis, that the combination of environmental conditions and storage conditions would not significantly influence TBS within the same pretreatment method, was rejected. Comparison of the mean TBS of the different environmental conditions in the MEP pretreatment group showed a similar pattern for 24 h or TC. For both storage conditions, a significant decrease in TBS was observed in specimens under OC compared to samples under RC and LC (Fig 2). We suggest that the high humidity level (RH 95%) was the main factor for the decrease in TBS under OC for the MEP and GBL pretreatment methods. This assumption is supported by Miyazaki et al,^47^ who reported a decrease in resin composite bond strengths to dentin using self-etching primer systems when RH exceeded a critical level of 80%. Those authors hypothesized that a perfect seal between the resin composite and the pretreated surface may be impaired by water droplets at the interface due to higher humidity levels.^47^ This mechanism seems applicable to the present study, where water droplets were also found in the OC groups (Fig 6). Miyazaki et al^47^ further concluded that supplementary humidity may interfere with the polymerization process and lead to an insufficiently polymerized resin coat remaining on the adhesive surface, which caused lower bond strengths. This effect may have occurred in the OC groups of the present study due to the high humidity levels.

There was no significant difference in mean TBS between LC and RC in the MEP group for either storage condition. LC and RC were established with the same humidity of 50% but with different temperatures (LC: 23°C; RC: 30°C), which suggests that a temperature change of 7°C has less influence on the mean TBS than differences in humidity. Several studies reported that temperature changes influenced the viscosity and polymerization shrinkage of composites.^6,30^ However, a significant effect may only occur for temperatures above 35°C. Temperature values between 23°C and 30°C induced only slight changes in composite shrinkage.^6,40,67^ Therefore, low-level temperature changes may be the decisive factor for non-significant alterations in mean TBS between LC and RC within the MEP pretreatment group. The effects of additional water from humidity on resin bonding materials may otherwise only be observed for high humidity values. Jacobsen et al^29^ demonstrated that a limited interaction between bis-GMA molecules and high water content did not significantly affect the polymerisation process of resin bond materials. They considered the limited interaction between water and bis-GMA monomers to be a key reason for the lack of effect on the curing degree of the bis-GMA molecules. This finding is consistent with our results, where the same humidity levels (50% RH) were achieved for LC and RC. As mentioned previously, the significant effects of humidity levels above 50% may be due to the influence of moisture on pretreatment materials but not on the resin bond material used in our study. Because the mean TBS of RC closely resembled the mean TBS of LC at the same humidity level, we suggest that the increased humidity was the main reason for the reduction in TBS seen in the experiments under OC. The present in vitro study indicated that low humidity led to a higher TBS of resin composite to LDS when pretreated with GBL or MEP. The literature shows that the use of rubber-dam clinically reduces intraoral humidity.^30,52^ These findings suggest that application of rubber-dam for intraoral glass-ceramic repair is recommended, but further in vivo studies are needed to verify these assumptions.

However, bonded areas inside the oral cavity must resist higher temperatures and humidities as well as material fatigue due to temperature changes and chemical factors provoked by acidic agents from oral fluids, food, and beverages.^7^ Thermocycling simulates this chemical and mechanical stress, and is a widely used in vitro accelerated aging protocol for bond stability.^15,58^ During the thermocycling process, the adhesive interface must withstand the increased hydrolysis of resin polymers induced by hot water and thermal stress, which cause repetitive expansion and contraction at the interfaces of the bonded materials.^5,58^ Asiry et al^5^ further proposed that the hydrolysis of the silane coupling agent at the adhesive interface was the key reason for the decrease in TBS and concluded that the silane-promoted adhesion diminished with longer exposure to the oral environment. Different expansion coefficients of the disilicate ceramic and resin additionally lead to fatigue of the materials, which decreases bonding durability and results in separation of the bonded ceramic blocks.^34^ This finding is consistent with our study, where TC significantly reduced the mean TBS of ceramic-resin bonds for all environmental conditions independent of the pretreatment method. The only exception was the MEP pretreatment group under OC, where no statistically significant difference was observed between the thermocycled and 24-h storage specimens (Fig 2). The smoother surface etching pattern generated by MEP is probably less prone to thermal stresses than the rough GBL surface, which has a higher probability of subsurface damage.^61^

Contrary to the findings in the MEP pretreatment group, the GBL group showed significantly reduced TBS in the RC compared to the LC groups after thermocycling (Fig 3). The slight changes in temperature and the weakening influence of GBL and TC may be sufficient to exceed the material’s bonding capacities and reduce TBS. In addition to the hydrolytic degradation during TC, we assumed that the GBL-pretreated interfaces were already weakened via crack propagation induced by the grit-blasting procedure itself, which made these samples more sensitive to thermally induced stress by TC.^66^ This weakening resulted in a more pronounced reduction in mean TBS and a remarkably higher number of PTFs than the MEP OC group.

The simulation of intraoral conditions in the present study revealed a significant impact on bond strengths for both pretreatment methods, and high temperature and high humidity influenced the chemical properties of the investigated materials. Notably, the simulated oral conditions improve the translation of laboratory research to dental practice. However, these results must be validated in a clinical study.

## Conclusion

- MEP pretreatment is a possible alternative to GBL for the repair of lithium-disilicate glass-ceramics;
- Increased humidity significantly reduced the adhesion potential of resin composites to lithium-disilicate glass-ceramics pretreated with MEP and GBL;
- Minimizing humidity is favorable for the adhesion of resin composite repairs to lithium-disilicate glass-ceramic, and the clinical use of a rubber-dam is suggested.

## Clinical relevance:

Minimizing moisture promotes the adhesion of resin composite repairs to lithium-disilicate glass-ceramic. Therefore, the use of rubber-dam in clinical practice is suggested, especially when pretreating the ceramic with grit-blasting.
